# SARS-CoV-2 airborne infection probability estimated by using indoor carbon dioxide

**DOI:** 10.1007/s11356-023-27944-9

**Published:** 2023-06-07

**Authors:** Narumichi Iwamura, Kanako Tsutsumi

**Affiliations:** Sasebo Chuo Hospital, 15, Yamato-Cho, Sasebo-Shi, Nagasaki 857-1165 Japan

**Keywords:** SARS-CoV-2, COVID-19, Airborne transmission, Wells-Riley model, CO2, Ventilation, Infection probability, R0

## Abstract

Airborne transmission is one of the main routes of SARS-CoV-2 spread. It is important to determine the circumstances under which the risk of airborne transmission is increased as well as the effective strategy to reduce such risk. This study aimed to develop a modified version of the Wells-Riley model with indoor CO_2_ to estimate the probability of airborne transmission of SARS-CoV-2 Omicron strains with a CO_2_ monitor and to evaluate the validity of this model in actual clinical practices. We used the model in three suspected cases of airborne transmission presented to our hospital to confirm its validity. Next, we estimated the required indoor CO_2_ concentration at which R_0_ does not exceed 1 based on the model. The estimated R_0_ (R_0_, basic reproduction number) based on the model in each case were 3.19 in three out of five infected patients in an outpatient room, 2.00 in two out of three infected patients in the ward, and 0.191 in none of the five infected patients in another outpatient room. This indicated that our model can estimate R_0_ with an acceptable accuracy. In a typical outpatient setting, the required indoor CO_2_ concentration at which R_0_ does not exceed 1 is below 620 ppm with no mask, 1000 ppm with a surgical mask and 16000 ppm with an N95 mask. In a typical inpatient setting, on the other hand, the required indoor CO_2_ concentration is below 540 ppm with no mask, 770 ppm with a surgical mask, and 8200 ppm with an N95 mask. These findings facilitate the establishment of a strategy for preventing airborne transmission in hospitals. This study is unique in that it suggests the development of an airborne transmission model with indoor CO_2_ and application of the model to actual clinical practice. Organizations and individuals can efficiently recognize the risk of SARS-CoV-2 airborne transmission in a room and thus take preventive measures such as maintaining good ventilation, wearing masks, or shortening the exposure time to an infected individual by simply using a CO_2_ monitor.

## Introduction

Severe acute respiratory syndrome coronavirus 2 (SARS-CoV-2) is a novel coronavirus that originated in Wuhan, Hubei Province, China, in December 2019 (Guan et al. 2020). The infection caused by this virus, referred to as coronavirus disease 2019 (COVID-19), has since spread worldwide. The initial symptoms of COVID-19 resemble those of influenza or the common cold and are difficult to distinguish in the early stages of the disease. The incubation period from SARS-CoV-2 exposure to disease onset is 1-14 days, usually 5 days. COVID-19 is contagious even before its onset and is even more highly contagious shortly after the onset of symptoms. Its high infectivity and asymptomatic nature in the early period following onset are believed to be the causes of community-acquired infections, which make it difficult to control its spread. The Japanese government has decided to classify COVID-19 as category 5 infectious disease, similar to seasonal flu, starting on May 8, 2023. However, facilities such as hospitals and clinics need to continue working on infection prevention as they cater to many patients who may easily get infected and be severely affected by acute respiratory distress syndrome and multiorgan failure, which may result in death. We observed COVID-19 patients who visited our hospital from April 4 to 18, 2022 to determine the severity of this disease in another study. In this study, 38.1% (223/584) of the infected patients were classified as moderate and severe cases (Iwamura et al. 2003**)**. This result suggests that even if COVID-19 is to be classified as category 5 infectious disease, several patients are still likely to be severely affected. Thus, the prevention of SARS-CoV-2 infection is still important.

The efficacy rate of the COVID-19 vaccine is reportedly around 90% (Thompson et al. 2021). However, with the emergence of new strains, the effect of the vaccine may possibly diminish. It has also been reported the vaccine caused aberrant immune response in some cases. We actually experienced a case of a healthy individual who developed hypocomplementemic urticarial vasculitis with hemophagocytic lymphohistiocytosis after receiving the vaccine (Iwamura et al. 2003). Thus, we should not rely solely on vaccination to prevent COVID-19. We should also take measures to break off the infection routes, including airborne transmission.

Respiratory viruses such as SARS-CoV-2 are typically transmitted through contact, droplets or aerosols (Comber et al. 2021). Contact transmission can occur directly through the hands of an infected person or indirectly through the presence of virus particles on inanimate objects known as fomites. Droplet transmission occurs when an infected individual produces large quantities of respiratory droplets containing virus particles by coughing, sneezing, or talking. In such situations, the virus can infect a susceptible individual through the mouth, nose, and eyes, however, this mode of transmission typically requires close contact so that the virus particles have a relatively short distance to travel before being deposited on the ground or surrounding surfaces. Airborne transmission, defined as the spread of infectious agents known as droplet nuclei through aerosols, differs from droplet transmission as the virus particles have smaller sizes, travel longer distances, and can float in the air for extended periods of time. The concentration and particle size of aerosols can vary with the activity in question (e.g., breathing, talking, coughing or sneezing).

Coccia 2021 investigated the association of wind speed or air pollution with the spread of COVID-19. They concluded that air pollution is more likely to occur in cities with low wind speed and that COVID-19 tends to spread more easily in those cities. The author assumed that weak wind keeps the virus tend particles floating in the air, resulting in airborne transmission **(**Coccia 2021a, b). It has also been reported that not only air pollution but also weather conditions play a role in the spread of COVID-19 (Núñez-Delgado et al. 2021). Moritz et al. investigated the influence of ventilation rates and the use or non-use of masks on SARS-CoV-2 infection rates at mass gatherings. They reported that higher ventilation rates were associated with lower SARS-CoV-2 positivity and hospitalization rates, although no significant differences were observed (Moritz et al. 2021).

Despite the fact that hand sanitization with alcohol and the use of surgical masks and face shields are regularly practiced in most hospitals to prevent contact and droplet infections, respectively, nosocomial SARS-CoV-2 infections have continued to occur, with some clusters of infections also being observed. In our hospital, we have also experienced several cases of nosocomial SARS-CoV-2 infection cases that was transmitted through the airborne route, two of which are described below. In the first case (details provided below), all medical personnel in the pediatric outpatient consultation room were infected despite wearing surgical masks and face shields. In the second case, all patients staying in the same ward as the infected person were infected despite the absence of any form of communication or contact between them and the isolation with the help of medical curtains. These are only representative cases of infection, in reality, there are many other cases caused by airborne transmission. Considering these cases, we inferred that, in addition to the contact and droplet routes, airborne transmission of SARS-CoV-2 may also occur. Therefore, all modes of transmission should be considered when establishing prevention strategies.

Measures such as hand sanitization with alcohol and the use of surgical masks and face shields to prevent contact and droplet transmissions are becoming increasingly common in most hospitals; however, measures such as maintaining good ventilation by opening windows and doors and wearing N95 masks to prevent airborne transmission are less practiced. This is mainly because the ventilation in hospitals reduces the efficacy of air conditioning systems, consequently increasing utility costs. Therefore, it is important to evaluate the conditions under which the risk of airborne infection is high and to identify the measurements (number, duration, frequency and size of windows and doors that should be opened) to reduce it. Jones et al. proposed a model to assess the relative risk of exposure to SARS-CoV-2; however, this model but this model requires air change and airflow rates, which are affected by room volume, room structure, air conditioning, ventilation fan, and wind speed, making it impractical (Jones. 2021). The Wells-Riley model is a conventional model used to estimate the probability of airborne transmission; however, predicting the indoor ventilation rate required for this model is difficult as it is largely dependent on the room structure, air velocity, air volume, performance of air conditioners and ventilation fans, and frequency of opening doors and windows. Adzic et al. monitored changes in CO_2_ concentration and investigated the association between CO_2_ concentration and the RNA copy number of SARS-CoV-2 (Adzic et al. 2022). Di Gilio et al. (2021) monitored the CO_2_ concentration inside educational buildings to evaluate the risk of COVID-19. They categorized the risk of infection using the CO_2_ concentration and listed the actions to be taken in each category; < 700 ppm (low risk), no action; < 800 ppm (moderate risk), door always open; < 1000 ppm (high risk), door always open and windows open for 10 min at midday; and 1000 ppm (very high risk), door always open and windows open for 10 min at the end of the teaching hour. Zhao et al. (2023) reported that in the absence of source other than the metabolic activity of humans inside the room, the CO_2_ concentration can reflect breathing and ventilation conditions. We considered the use of indoor CO_2_ concentration as an indicator of ventilation to predict airborne transmission. Therefore, the present study aimed to predict the probability of airborne transmission using indoor CO_2_ concentration measured using a CO_2_ monitor.

## Materials and methods

### Sample and data

Samples of measurement results of CO_2_ concentration are measured in Sasebo Chuo Hospital.

### Measures of parameters

CO_2_ concentration was measured using a nondispersive-infraRed system CO_2_ recorder, TR-76Ui. Atmospheric CO_2_ concentration was measured every 30 s for 60 min on the rooftop of Sasebo Chuo Hospital on January, 2023. We adopted the median values of all data after reaching a plateau as atmospheric CO_2_ concentration.

Indoor CO_2_ concentration was measured every 30 s for 8-180 min in the pediatric outpatient room, ward, and respiratory outpatient room at Sasebo Chuo Hospital on January 2023. We adopted the median values of all data after reaching a plateau as indoor CO_2_ concentration.

### Models and data analysis procedure

Riley et al. (1978) developed the Wells-Riley equation to estimate the probability of airborne transmission of infectious agents indoors:

1 $${\boldsymbol{P}}_{\boldsymbol{I}}=\frac{\boldsymbol{C}^{\prime }}{\boldsymbol{S}}=1-{\boldsymbol{e}}^{-\frac{\boldsymbol{I}\boldsymbol{q}\boldsymbol{p}\boldsymbol{t}}{\boldsymbol{Q}}}$$

where

**P**_**I**_ = infection probability (−)

**C'** = number of susceptible individuals that were infected (−)

**S** = number of susceptible individuals (−)

**I** = number of infectious individuals (−)

**q** = generation rate of infectious quanta (/h)

**p** = pulmonary ventilation rate of a person (m^3^/h)

**t** = exposure time (h)

**Q** = rate of room ventilation with clean air (m^3^/h)

The Wells-Riley model has a prerequisite that the air in the room should be well mixed to enable uniform distribution of the aerosols are uniformly distributed, suggesting that it considers airborne transmission but neither droplet nor contact transmission. In addition, the model does not consider whether infectious particles are activated or nonactivated.

Since the start of the SARS-CoV-2 pandemic, the majority of the Japanese population have used face masks. Whether infectious and susceptible individuals wear a mask or not is important in the estimation of the infection probability, as well as the type of mask used (e.g., surgical or N95). Dai and Zhao (2020) proposed a modified version of the Wells-Riley model (shown below), which takes these factors into consideration.

2 $${\boldsymbol{P}}_{\boldsymbol{I}}=\frac{{\boldsymbol{C}}^{\prime }}{\boldsymbol{S}}=\textbf{1}-{\boldsymbol{e}}^{-\frac{\boldsymbol{I}\boldsymbol{qpt}}{\boldsymbol{Q}}\left(\textbf{1}-{\boldsymbol{\eta}}_{\boldsymbol{I}}\right)\left(\textbf{1}-{\boldsymbol{\eta}}_{\boldsymbol{S}}\right)}$$

where

**η**_**I**_ = exhalation filtration efficacy (−)

**η**_**S**_ = respiration filtration efficacy (−)

The Wells-Riley model is often difficult to use as it requires the rate of room ventilation with clean air (Q), which was previously estimated considering the performances of ventilation fans and air conditioners. . However, in reality, the ventilation rate is influenced by the presence of a door, and a window as well as the speed and direction of the wind outside, rendering accurate estimation difficult and thus preventing the use of the Wells-Riley model. Therefore, in the present study, the room ventilation rate was estimated considering the indoor CO_2_ concentration and emission rate instead. The Seidel formula shown below presents the relationship between the room ventilation rate and the indoor CO_2_ concentration:

3 $${\displaystyle \begin{array}{c}{\boldsymbol{C}}_{\boldsymbol{O}}\bullet {\boldsymbol{Q}}^{\boldsymbol{\prime}}\bullet \boldsymbol{dt}+\boldsymbol{M}\bullet \boldsymbol{dt}-\boldsymbol{C}\bullet {\boldsymbol{Q}}^{\boldsymbol{\prime}}\bullet \boldsymbol{dt}=\boldsymbol{V}\bullet \boldsymbol{dC}\\ {}{\boldsymbol{C}}={\boldsymbol{C}}_{\boldsymbol{O}}+\left({\boldsymbol{C}}_{\boldsymbol{s}}-{\boldsymbol{C}}_{\boldsymbol{O}}\right){\boldsymbol{e}}^{-\frac{\boldsymbol{Q}}{\boldsymbol{V}}\boldsymbol{t}}+\frac{\boldsymbol{M}}{\boldsymbol{Q}}\left(1-{\boldsymbol{e}}^{-\frac{\boldsymbol{Q}}{\boldsymbol{V}}\boldsymbol{t}}\right)\end{array}}$$

where

**C** = indoor CO_2_ concentration (ppm)

***C***_***O***_ = atmospheric CO_2_ concentration (ppm)

***C***_***S***_ = initiation value of the indoor CO_2_ concentration (ppm)

***Q'*** = rate of room ventilation with clean air per person (m^3^/h/person)

**V** = room volume (m^3^)

**T** = time (h)

**M** = CO_2_ emission rate of a person (m^3^/h/person)

If the CO_2_ emission rate of a person (M) is constant and sufficient time has passed, the indoor CO_2_ concentration (C)

stabilizes. Therefore, the rate of room ventilation with clean air per person (*Q*^′^) is as follows:

4 $${\displaystyle \begin{array}{c}\textbf{C}={\boldsymbol{C}}_{\textbf{0}}+\frac{\boldsymbol{M}}{{\boldsymbol{Q}}^{\prime }}\\ {}{\textbf{Q}}^{\prime }=\frac{\boldsymbol{M}}{\boldsymbol{C}-\boldsymbol{Co}}\end{array}}$$

where

***Q***^**′**^= rate of room ventilation with clean air per person (m^3^/h/person)

**C** = indoor CO_2_ concentration (ppm)

**Co** = atmospheric CO_2_ concentration (ppm)

**M** = CO_2_ emission rate of a person (m^3^/h/person)

The rate of room ventilation with clean air (Q) is as follows:

5 $${\boldsymbol{Q}}={\boldsymbol{n}\boldsymbol{Q}}^{'}$$

where

**Q** = rate of room ventilation with clean air (m^3^/h)

**n** = number of individuals staying in the room (person)

The rate of room ventilation with clean air (Q) can be estimated by substituting Eq. (5) for Eq. (4) as follows:

6 $${\boldsymbol{Q}}=\boldsymbol{n}\frac{\boldsymbol{M}}{\boldsymbol{C}- \boldsymbol{Co}}$$

The modified Wells-Riley model with indoor CO_2_ (C) can be obtained by substituting Eq. (2) for Eq. (6):

7 $${\boldsymbol{P}}_{\boldsymbol{I}}=\frac{\boldsymbol{C}^{\boldsymbol{\prime} }}{\boldsymbol{S}}=\boldsymbol{1}-{\boldsymbol{e}}^{\left\{-\frac{\boldsymbol{C}- \boldsymbol{Co}}{\boldsymbol{M}}\bullet \frac{\boldsymbol{Iqpt}}{\boldsymbol{n}}\left(\boldsymbol{1}-{\boldsymbol{\eta}}_{\boldsymbol{I}}\right)\left(\boldsymbol{1}-{\boldsymbol{\eta}}_{\boldsymbol{S}}\right)\right\}}$$

8 $${{\boldsymbol{R}}}_{\boldsymbol{0}}=\frac{{\boldsymbol{S}}}{{{\boldsymbol{P}}}_{{\boldsymbol{I}}}}$$

where

**P**_**I**_ = infection probability (−)

**C'** = number of susceptible individuals that were infected (−)

**S** = number of susceptible individuals (−)

**C** = indoor CO_2_ concentration (ppm)

**Co** = atmospheric CO_2_ concentration (ppm)

**M** = CO_2_ emission rate of a person (m^3^/h/person)

**I** = number of infectious individuals (−)

**q** = generation rate of infectious quanta (/h)

**p** = pulmonary ventilation rate of a person (m^3^/h)

**t** = exposure time (h)

**n** = number of individuals staying in the room (−)

**η**_**I**_ = exhalation filtration efficacy (−)

**η**_**S**_ = respiration filtration efficacy (−)

**R**_**0**_ = basic reproduction number

To determine the acceptable level for individual exposure risk from the perspective of public health perspective in which outbreaks need to be minimized, the basic reproduction number R_0_ was used. The basic reproduction number is defined as the expected secondary infections (S) caused by a typical infector (P_I_) among a completely susceptible population (R_0_ = S/P_I_). When R_0_ > 1, the virus may spread among the population, thus, so the target level of exposure risk is set to R_0_ < 1 (Yan et al. 2022).

The parameters of the modified Wells-Riley model with indoor CO_2_ are indoor CO_2_ concentration (C), atmospheric CO_2_ concentration (C_0_), CO2 emission rate for a person (M), number of infectious individuals (I), generation rate of infectious quanta (q), pulmonary ventilation rate for a person (p), exposure time (t), number of individuals staying in the room (n), exhalation filtration efficacy (η_I_), and respiration filtration efficacy (η_s_) (Table 1).

**Table 1:** Parameters of the modified Wells-Riley model with indoor CO_2_

Inputs	Unit	Value	References
Indoor CO_2_ concentration (C)	ppm	549-1187	measured using the CO_2_ recorder TR-76Ui
Atmospheric CO_2_ concentration (C_0_)	ppm	463	measured using the CO_2_ recorder TR-76Ui
CO_2_ emission rate for a person (M)	m^3^/h	0.011 at rest, 0.01795 for talking	Tajima et al. 2016
Number of infectious individuals (I)		1	
Generation rate of infectious quanta (q)	/h	20 for respiration, 1535 for conversation	Wang et al. 2023 and Dai and Zhao 2023
Pulmonary ventilation rate for a person (p)	m^3^/h	0.48	Liao et al. 2005
Exposure time (t)	hours	0.25-48	
Number of individuals staying in the room (n)		3-5	
Exhalation filtration efficacy (η_I_)		0 for no mask, 0.5 for surgical mask, 0.9 for N95 masks	Sickbert-Bennett et al. 2022
Respiration filtration efficacy (η_S_)		0 for no mask, 0.5 for surgical masks, 0.9 for N95 masks	Sickbert-Bennett et al. 2022

The indoor CO_2_ concentration(C) was set at 1000 ppm which is generally considered to be the cut-off point that distinguishes between good and bad ventilation in Japan, whereas the atmospheric CO_2_ concentration (*C*_*O*_) was set at 463 ppm based on our measurements (Table 2).

**Table 2:** Atmospheric and indoor CO_2_ concentration

	Atmospheric CO_2_	Indoor CO_2_ concentration		
Location	Rooftop of our hospital	Pediatric outpatient room	Ward	Respiratory outpatient room
Date	January 6, 2023 11:37-12:37	January 6, 2023 17:30-18:00	January 6, 2023 3:00-6:00	January 13, 2023 18:22-18:30
Time	1 hour	30 minutes	3 hours	8 minutes
Interval	30 seconds	30 seconds	30 seconds	30 seconds
Number of data points	121	62	362	17
Instrument	CO_2_ Recorder TR-76Ui			
Temperature; median [Q1, Q3] *	10.6 [10.4, 11.1]	25.2 [25.1, 25.2]	25.3 [25.2, 25.5]	22.65 [22.6, 22.7]
Humidity; median [Q1, Q3] *	61.0 [60.0, 61.2]	28.0 [28.0, 29.0]	26.0 [26.0, 26.0]	68.5 [68.0, 69.0]
CO_2_ concentration; median [Q1, Q3] *	463 [458, 466]	1116 [1100, 1130]	1187 [1177, 1193]	549 [546, 552]

*Q1, first quartile, Q3, third quartile

Tajima et al. (2016) found that the CO_2_ emission rate of a male adult (M) ranges between 0.011 and 0.0840 based on the work intensity as follows, 0.011 at rest; 0.0129-0.0230 doing paperwork while sitting; 0.0230-0.0330 walking slowly; 0.0330-0.0538 light labour; 0.0538-0.0840 moderate labour; and >0.0840 heavy labour. They suggested that multiplying these values by 0.9 for a female adult and by 0.5 for a child for correction (Tajima et al. 2016). Based on this study, we set the CO_2_ emission rate of a male adult as follows, 0.0110 at rest; 0.01795 doing papaerwork while sitting; 0.0280 walking slowly; 0.0434 light labour; 0.0689 moderate labour; 0.0840 heavy labour. Multiplying these values by 0.9 for a female adult and 0.5 for a child was suggested (Table 3).

**Table 3:** CO_2_ emission rate for each working level

Relative metabolic rate	Working level	CO_2_ emission rate [m^3^/h] (Tajima et al. 2016)	Average of CO_2_ emission rate [m^3^/h]
0	At rest	0.011*	0.0110
0.0-1.0	Doing paperwork while sitting	0.0129-0.0230*	0.01795
1.0-2.0	Walking slowly	0.0230-0.0330*	0.0280
2.0-4.0	Light labour	0.0330-0.0538*	0.0434
4.0-7.0	Moderate labour	0.0538-0.0840*	0.0689
> 7.0	Heavy labour	> 0.0840*	0.0840

*Female, multiply the table value by 0.9; children, multiply the table value by 0.5

An infectious quantum is a hypothetical unit of infectivity calculated from epidemiological studies indicating the assembly of viral particles required to establish infection. Prentiss et al. (2022) previously estimated the generation rate of infectious quanta (q) using the Wells-Riley model on six super-spreader cases in the early stage of COVID-19 pandemic. The authors reported that the estimated infectious quanta emitted through speaking ranged from 136 to 757 /h, with the average being 461 /h. They have also reported that the estimated infectious quanta emitted through breathing ranged from 3 to 17 /h, with the average beiing10 /h. Based on another report by Dai and Zhao (2023), the infectious quanta was estimated from14 to 48 /h. In the follow-up report, the authors mentioned the possibility of infectious quanta varying from mutant strains of SARS-CoV-2. They obtained the generation rate of infectious quanta (q) of three SARS-CoV-2 variants (Alpha, Delta, and Omicron) for the Wells―Riley equation with a reproductive number-based fitted approach and estimated the association between the infection probability and ventilation rates (Dai and Zhao H 2023**)**. Based on this study, the value of q was 89-165 /h for Alpha, 312-935 /h for Delta, and 725-2345 /h for Omicron. We set the q value emitted through speaking at 1535 /h, which value is the median of minimum and maximum q value for Omicron variant. On the other hand, Wang et al. (2023) have evaluated the infection probability of SARS-CoV-2 in different types of aircraft cabins using the Wells-Riley model. In the study, 20 /h was used as the q value. We set the q value emitted through breathing at 20 /h based on this study. The pulmonary ventilation rate of a person (p) is usually 0.48 m^3^/h (Liao et al. 2005).

Sickbert-Bennett et al. (2022) examined the filtration efficacy of a hospital face mask and found that N95 masks with suboptimal fit still had a comparable efficacy of >90%. Surgical masks secured with either ties or ear loops had a much lower filtration efficacy of 37% to 69%, as might be expected from their thinner filters and looser fit. In the presemt study, the exhalation filtration efficacy (η_I_) and respiration filtration efficacy (η_S_) were set at 0% for no mask, 50% for surgical masks and 90% for N95 masks.

Aganovic et al. investigated the relationship between humidity (20%, 37%, 53%, and 70%) and the risk of SARS-CoV-2 infection (Aganovic et al. 2022). With the artificial medium, the risk of infection was the highest at a humidity of 20%, followed by 37%, 53%, and then 70%. With the culture medium, the risk of infection was the highest at a humidity of 53%, followed by 20%, 37% and then 70%. Based on these results, the authors concluded that the relationship between humidity and the risk of SARS-CoV-2 infection is unclear. Therefore, we considered it is not necessary to consider humidity in estimating the probability of SARS-CoV-2 infection.

Coccia (2022) demonstrated that high levels of vaccination can reduce case fatality rations and mortality rates of COVID-19 associated with other factors, namely, health, environmental, and economic systems. As they mentioned, the SARS-CoV-2 vaccine can help reduce disease severity and the rate of mortality caused by the disease, however, it remains unclear whether the vaccine can help reduce infection probability. Therefore, we found that it is unnecessary to consider vaccination in estimating the infection probability of SARS-CoV-2.

## Results and discussion

### Cases of airborne transmission

The modified Wells-Riley model with indoor CO_2_ was used in two suspected cases of SARS-CoV-2 airborne transmission and one case of non-infection in our hospital to confirm its validity and determine its limitations.

#### In a pediatric outpatient room

A 1-year-old boy was brought by his mother to the pediatric consultation room in our hospital for a periodic medical check-up on July 15, 2022. During that time, SARS-CoV-2 BA.5 was prevalent in Japan, although no nosocomial infections had been recorded. The patient had fever and was crying profusely. Five individuals were present in the room including the patient, his mother, a pediatrician, a nurse and a medical intern. The patient’s mother wore a surgical mask, but he himself did not have any mask. Neither of them wore face shields. The pediatrician, nurse and medical intern wore surgical masks and face shields, and none of them had a history of SARS-CoV-2 infection or any other underlying diseases. The outpatient consultation room had no window but had three doors, all of which were kept closed. The consultation lasted about 30 min. Two days later, the pediatrician, nurse, and medical intern had a slight fever and sore throat and later tested positive for SARS-CoV-2 via PCR test. Everyone else who had also come into contact with them tested negative for SARS-CoV-2. A few days later, the boy’s mother informed us via phone that he had been infected with SARS-CoV-2.

Although impossible to prove, it is highly likely that SARS-CoV-2 was transmitted from the boy to the healthcare workers inside the room given the surrounding conditions. Furthermore, the primary route of transmission seemed to be airborne as all the infected healthcare workers wore surgical masks and face shields.

The probability of airborne infection was estimated using the modified Wells-Riley model with indoor CO_2_ to identify the prevention measures that should have been implemented. The indoor CO_2_ concentration under nearly identical circumstances (five people in the same consultation room with all three doors closed) was measured and set at 1116 ppm (Table 2). The infection probability and R_0_ were estimated to be 79.7% and 3.19, respectively, which were nearly identical to the number of individuals actually infected in this case (3). The parameters of this model were as follows, indoor CO_2_ concentration (C), 1116 ppm; atmospheric CO_2_ concentration (C_O_), 463 ppm; CO_2_ emission rate for a person, 0.015078 [=0.01795*(0.5*1 + 0.9*3 + 1*1)/5]; number of infectious individuals (I), 1; generation rate of infectious quanta, 1535/h; pulmonary ventilation rate for a person (p), 0.48 m^3^/h; exposure time (t), 0.5 hours; number of individuals staying in the room (n), 5; exhalation filtration efficacy (η_**I**_), 0; and respiration filtration efficacy (η_S_), 0.5**.**

**Fig. 1:**
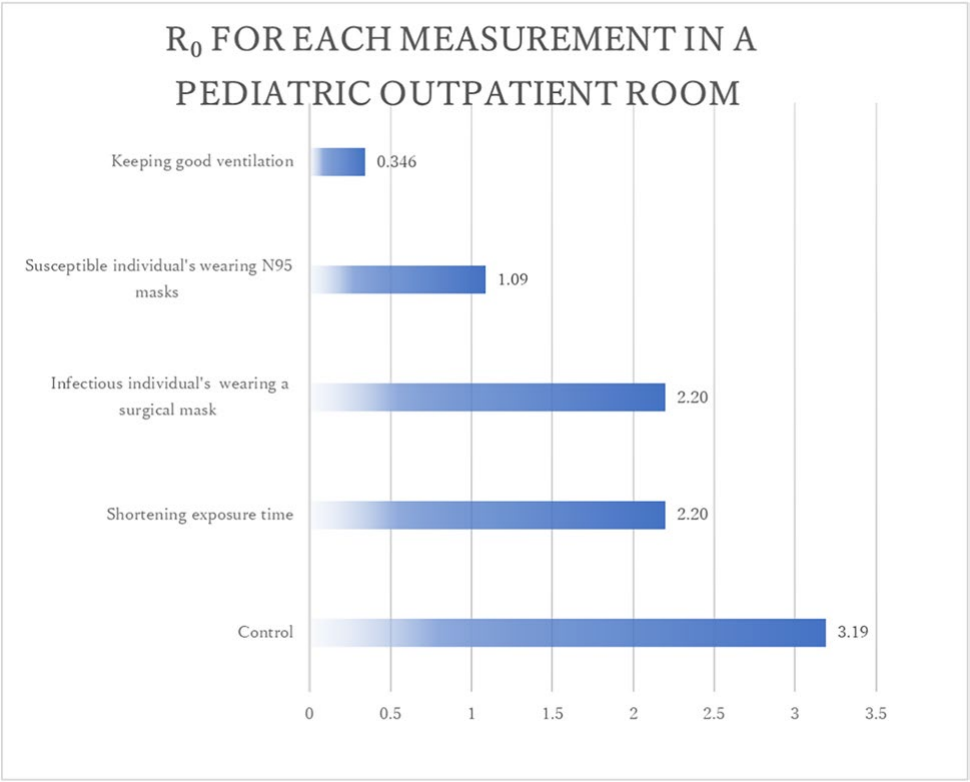
R_0_ for each measurement in the pediatric outpatient room estimated by the modified Wells-Riley model with indoor CO_2_

Furthermore, we identified the effective measurements for reducing infection probability and R_0_ based on the modified Wells-Riley model with indoor CO_2_. When the exposure time was shortened from 0.50 to 0.25 h, the infection probability and R_0_ decreased from 79.7% to 55.0% and from 3.19 to 2.20, respectively. When the patient wore a surgical mask instead of no mask, the infection probability and R_0_ decreased from 79.7% to 55.0% and from 3.19 to 2.20, respectively. When the susceptible individual wore an N95 mask instead of a surgical mask, the infection probability and R_0_ significantly decreased from 79.7% to 27.3% and from 3.19 to 1.09, respectively. When the room was provided with good ventilation, with the indoor CO_2_ concentration being 500 ppm based on actual measurements which was achieved by opening all doors and windows, the infection probability and R_0_ significantly decreased from 79.7% to 8.64% and from 3.19 to 0.346, respectively (Fig. 1). These results suggest that maintaining good ventilation by keeping doors and windows open and wearing N95 masks can significantly reduce the risk of SARS-CoV-2 airborne transmission.

#### In a hospital ward

Three patients (A, B and C) were admitted to the ward in our hospital on August 29, 2022, during which SARS-CoV-2 BA.5 was still prevalent in Japan. None of the patients wore masks or conversed and the windows and doors in the ward were kept closed. The patients’ beds were separated by medical curtains.

Patient A was an 82-year-old woman receiving tofacitinib citrate 10 mg/day and prednisolone 5 mg/day for rheumatoid arthritis and Sjögren’s syndrome. She was admitted to our hospital for lumbar disc herniation and then transferred to another hospital where she tested positive for SARS-CoV-2 *via* PCR. Chest computed tomography revealed multiple ground-glass opacities, some of which were considered to have been present for several days after onset. Patients B and C also tested positive for SARS-CoV-2 thereafter. Patient B, an 80-year-old woman receiving prednisolone 5 mg/day and methotrexate 8 mg/week for rheumatoid arthritis, underwent humeral osteosynthesis for right humeral neck fracture. On the other hand, Patient C, a 72-year-old woman receiving methotrexate 8 mg/day for rheumatoid arthritis, underwent osteosynthesis for distal radius fracture. All healthcare workers who were exposed to them tested negative for SARS-CoV-2 via PCR.

Although impossible to prove, it is likely that SARS-CoV-2 was transmitted from Patient A to the other two patients in the same ward. Furthermore, the primary route of transmission seemed to be airborne as the patients in the ward did not talk to each other and their beds were separated by curtains.

The indoor CO_2_ concentration was measured under nearly identical circumstances (four people in the same ward, with the doors and windows kept closed), and was set at 1187 ppm (Table 3). The probability of airborne infection and R_0_ was estimated to be 99.6% and 1.99, respectively, which were nearly identical to the actual numbers of individuals infected in this case (2). The parameters of this model were as follows, indoor CO_2_ concentration (C), 1187 ppm; atmospheric CO_2_ concentration (C_O_), 463 ppm; CO_2_ emission rate for a person, 0.0099 (= 0.011*0.9) m^3^/h; number of infectious individuals (I), 1; generation rate of infectious quanta, 20/h; pulmonary ventilation rate of a person (p), 0.48 m^3^/h; exposure time (t), 24 hours; number of individuals staying in the room (n), 4; exhalation filtration efficacy (η_**I**_), 0; and respiration filtration efficacy (η_S_) , 0.

**Fig. 2:**
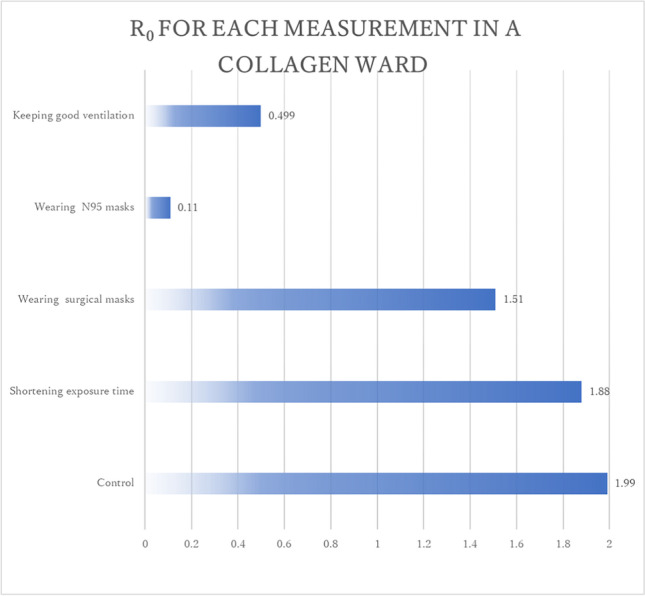
R_0_ for each measurement in the ward estimated by the modified Wells-Riley model with indoor CO_2_

When the exposure time was shortened from 24 to 12 h, the infection probability and R_0_ decreased from 99.6% to 94.0% and from 1.99 to 1.88, respectively. When all the patients in the ward wore surgical masks instead of no mask, the infection probability and R_0_ decreased to from 99.6% to 75.4% and from 1.99 to 1.51, respectively. Contrarily, when the patients wore N95 masks instead of no mask, infection probability and R_0_ significantly decreased from 99.6% to 5.5% and from 1.99 to 0.11, respectively. When the ward was provided with good ventilation, with the indoor CO_2_ concentration being 500 ppm, based on actual measurements, which was achieved by opening the door leading to the corridor, the infection probability and R_0_ is significantly decreased from 99.6% to 25% and from 1.99 to 0.499, respectively (Fig. 2).

These results, suggest that the risk of airborne transmission can be significantly reduced by wearing N95 masks inside the ward. However, it is impossible for all patients to wear N95 masks in the hospital. Therefore, maintaining good ventilation as much as possible is the feasible alternative measure to prevent airborne transmission in the ward.

#### In a respiratory outpatient room

A 74-year-old man made a routine visit to the respiratory clinic. He was asymptomatic on the day of presentation but tested positive for SARS-CoV-2 *via* PCR the following day. The examining physician, medical secretary, and two nurses, one of whom had been previously infected with COVID-19, were negative in the daily PCR testing. The patient wore a surgical mask, and all medical staff wore face shields and surgical masks. In the consultation room, two doors leading to the next room and all the windows were open, whereas the door leading to the corridor was closed.

We measured the indoor CO_2_ concentration under nearly identical conditions (five people in the same room, with two doors open) and all windows open. We set the indoor CO_2_ concentration at 549 ppm based on the measurement results.

The infection probability and R_0_ based on the modified Wells-Riley model with indoor CO_2_ were estimated to be 4.79% and 0.191, respectively, which were nearly identical to the number actually infected in this case (0). The parameters of this model were as follows, indoor CO_2_ concentration (C), 549 ppm; atmospheric CO_2_ concentration (C_O_), 463 ppm; CO_2_ emission rate for a person, 0.0162 ( = 0.01795*0.9); number of infectious individuals (I), 1; generation rate of infectious quanta, 1535/h; pulmonary ventilation rate for a person (p), 0.48 m^3^/h; exposure time (t), 15 minutes; number of individuals staying in the room (n), 5; exhalation filtration efficacy (η_**I**_), 0.5; and respiration filtration efficacy (η_S_), 0.5.

**Fig. 3:**
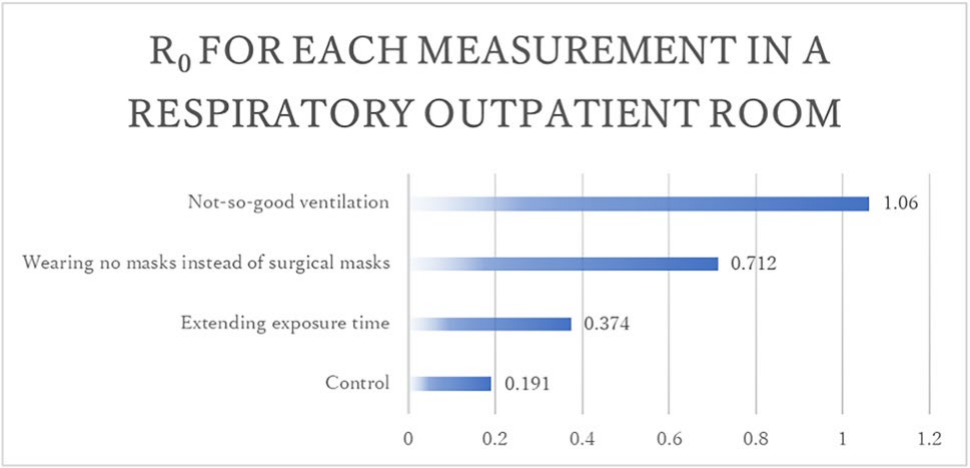
R_0_ for each measurement in the respiratory outpatient room estimated by the modified Wells-Riley model with indoor CO_2._ *Outpatient setting; the parameters of this model were as follows: atmospheric CO_2_ concentration (C_O_), 463 ppm; CO_2_ emission rate for a person, 0.01795 m^3^/h; number of infectious individuals (I), 1; generation rate of infectious quanta, 1535/h; pulmonary ventilation rate for a person (p), 0.48 m^3^/h; exposure time (t), 15 minutes; number of individuals staying in the room (n), 5

In this case, SARS-CoV-2 airborne transmission was prevented despite the fact that the susceptible individuals did not wear N95 masks during exposure the infected patient. We identified the effective measure to prevent airborne transmission. When the exposure time was extended from 15 to 30 min, the infection probability and R_0_ slightly increased from 4.79% to 9.34% and from 0.191 to 0.374, respectively. When both the infected and susceptible individuals wore no mask instead of wearing surgical masks, the infection probability and R_0_ increased from 4.79% to 17.8% and from 0.191 to 0.712, respectively. If an infected individual does not wear any mask, the infection probability would be higher because the main transmission route would be droplet. If the room is not well ventilated, with the indoor CO_2_ concentration being 1000 ppm, based on actual measurements, which could be achieved by closing all doors and windows inside the room, the infection probability and R_0_ is significantly increased from 4.79% to 26.4% and from 0.191 to 1.06, respectively (Fig. 3).

These results suggest that maintaining good ventilation is the most effective measure to prevent airborne transmission in an outpatient room.

### Permissible indoor CO_2_ concentration

We suggest permissible indoor CO_2_ concentration preventing airborne transmission based on the modified Wells-Riley model with indoor CO_2_ in each case of typical outpatient and impatient settings. We considered when P_I_ < 1%, SARS-CoV-2 airborne transmission can hardly occur, and when R_0_ < 1, the spread of SARS-CoV-2 through airborne transmission can hardly occur.

#### In an outpatient consultation room

**Fig. 4:**
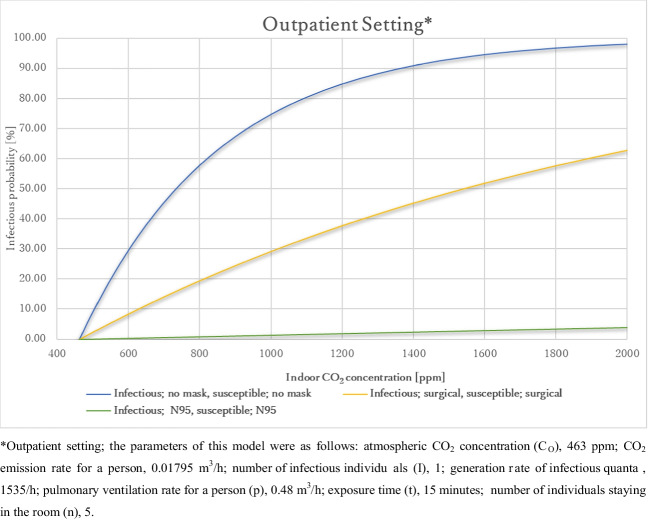
Relationship between the indoor CO_2_ concentration and infection probability in the outpatient setting. *Outpatient setting; the parameters of this model were as follows: atmospheric CO_2_ concentration (C_O_), 463 ppm; CO_2_ emission rate for a person, 0.01795 m^3^/h; number of infectious individuals (I), 1; generation rate of infectious quanta, 1535/h; pulmonary ventilation rate for a person (p), 0.48 m^3^/h; exposure time (t), 15 min; number of individuals staying in the room (n), 5. **Inpatient setting; parameters of this model were as follows: atmospheric CO_2_ concentration (C_O_), 463 ppm; CO_2_ emission rate for a person, 0.011 m^3^/h; number of infectious individuals (I), 1; generation rate of infectious quanta, 20/h; pulmonary ventilation rate for a person (p), 0.48 m^3^/h; exposure time (t), 24 h; number of individuals staying in the room (n). 4

Figure 4 presents the relationship between indoor CO_2_ concentration and the infection probability of airborne transmission in an outpatient consultation room under certain circumstances: there were four individuals in the room, a doctor, a nurse, a medical assistant, and an infected patient, talking to each other, the exposure time was 15 min. The parameters of this model were as follows: atmospheric CO_2_ concentration (C_O_), 463 ppm; CO_2_ emission rate for a person, 0.01795 m^3^/h; number of infectious individuals (I), 1; generation rate of infectious quanta , 1535/h; pulmonary ventilation rate for a person (p), 0.48 m^3^/h; exposure time (t), 15 minutes; number of individuals staying in the room (n), 5. The infection probability increased as the indoor CO_2_ concentration increased, and as expected, the infection probability was the highest when wearing no mask, followed by the use of surgical masks, and then N95 masks, at the same indoor CO_2_ concentration.

**Fig. 5:**
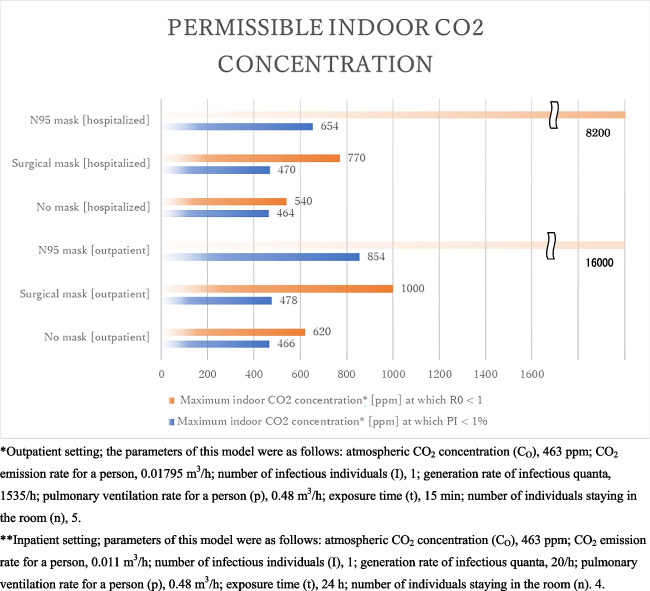
Permissible indoor CO_2_ concentration in a typical outpatient and inpatient settings. *Inpatient setting; parameters of this model were as follows: atmospheric CO_2_ concentration (C_O_), 463 ppm; CO_2_ emission rate for a person, 0.011 m^3^/h; number of infectious individuals (I), 1; generation rate of infectious quanta, 20/h; pulmonary ventilation rate for a person (p), 0.48 m^3^/h; exposure time (t), 24 h; number of individuals staying in the room (n), 4

Figure 5 presents the maximum indoor CO_2_ concentration at which P_I_ was below 1% and R_0_ was below 1. When both the infected and susceptible individuals wore no mask, the maximum indoor CO_2_ concentrations at which P_I_ < 1% and R_0_ < 1 were estimated to be 466 and 620 ppm, respectively. An indoor CO_2_ concentration of 466 ppm is nearly impossible to achieve; thus, completely preventing SARS-CoV-2 airborne transmission without the use of any mask is very difficult in an outpatient room. When both the infected and susceptible individuals wore surgical masks, the maximum indoor CO_2_ concentrations at which P_I_ < 1% and R_0_ < 1 were estimated to be 478 and 1000 ppm, respectively. These results suggest that preventing airborne transmission by wearing surgical masks is possible in an outpatient room with good ventilation. On the other hand, the spread of infection *via* airborne transmission can be prevented with well ventilation when people in an outpatient room wear surgical masks. When both the infected and susceptible individuals wore N95 masks, the maximum indoor CO_2_ concentrations at which P_I_ < 1% and R_0_ < 1 were estimated to be 854 and 16000 ppm. These results suggest when people wear N95 masks, airborne transmission of SARS-CoV-2 can be prevented with well ventilation. On the other hand, the spread of SARS-CoV-2 by airborne transmission hardly occur regardless of whether the ventilation is good or bad in an outpatient room.

However, this does not mean that merely keeping the indoor CO_2_ concentration below these values can prevent the spread of SARS-CoV-2 infection as this model does not consider droplet or contact transmission. account. Other measures, such as the use of face or eye shields, should be implemented to prevent droplet and contact transmissions.

#### In a hospital ward

**Fig. 6:**
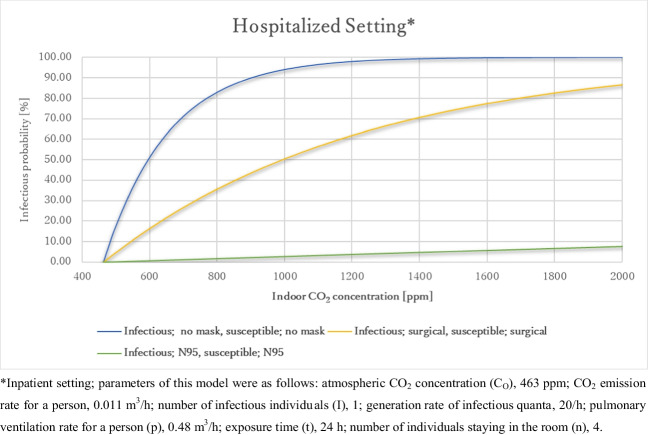
Relationship between indoor CO_2_ concentration and infection probability of SARS-CoV-2 in the inpatient setting

Figure 6 presents the relationship between indoor CO_2_ concentration and the infection probability of airborne transmission in an outpatient consultation room under certain circumstances, there were four patients in the room, and they were not talking to each other; the exposure time was 24 h. The parameters of this model were as follows: atmospheric CO_2_ concentration (C_O_), 463 ppm; CO_2_ emission rate for a person, 0.011 m^3^/h; number of infectious individuals (I), 1; generation rate of infectious quanta, 20/h; pulmonary ventilation rate for a person (p), 0.48 m^3^/h; exposure time (t), 24 h; number of individuals staying in the room (n), 4. The Infection probability increased as the indoor CO_2_ concentration increased, and as expected, the infection probability was the highest when wearing no mask, followed by the use of surgical masks, and then N95 masks at the same CO_2_ concentration.

Figure 5 presents the maximum indoor CO_2_ concentration at which P_I_ does not exceed 1% and R_0_ does not exceed 1. When both the infected and susceptible individuals wore no mask, the maximum indoor CO_2_ concentrations at which P_I_ < 1% and R_0_ < 1 were estimated to be 464 ppm and 540 ppm, respectively. These results suggest that without any mask it is nearly impossible for completely preventing airborne transmission and rather difficult for preventing spread of infection by airborne transmission of SARS-CoV-2 in a ward. This is because all doors and windows a need to be left open all night to keep the indoor CO_2_ concentration below 540 ppm, which is difficult to achieve because of the weather the weather or season. When both the infected and susceptible individuals wore surgical masks, the maximum indoor CO_2_ concentrations at which P_I_ < 1% and R_0_ < 1 were estimated to be 470 and 770 ppm, respectively. These results suggest that even if surgical masks are worn, it is nearly impossible for completely preventing airborne transmission and can be possible with good ventilation for preventing spread of infection by airborne transmission of SARS-CoV-2 in a ward. When both the infected and susceptible individuals wore N95 masks, the maximum indoor CO_2_ concentrations at which P_I_ < 1% and R_0_ < 1 were estimated to be 654 and 8200 ppm, respectively. These results suggest that when people wear N95 masks, airborne transmission of SARS-CoV-2 can be prevented with good ventilation. On the other hand, the spread of SARS-CoV-2 by airborne transmission can hardly occur regardless of whether the ventilation is good or bad in a ward. However, it is practically impossible for all hospitalized patients to wear N95 masks all night. Therefore, we conclude that wearing surgical masks as long as possible, in addition to maintaining good ventilation with an indoor CO_2_ concentration of less than 770 ppm, can effectively prevent the spread of SARS-CoV-2 through airborne transmission in a hospital ward. Based on these results, maintaining good ventilation is indeed important to prevent airborne infection in most cases. Ventilation strategies should be adopted decrease the time of SARS-CoV-2 residence, which is related to the time taken to reach a steady state number of RNA copies in a space (Jones et al. 2021). To achieve this, we recommend that as many windows and doors be kept open as possible. Maintaining good ventilation during the hot season is particularly important because the ventilation rates are often low during this period, while both of the risks of SARS-CoV-2 airborne transmission and the benefits of thermal comfort of occupants and heating energy demand need to be considered (Jones et al. 2021).

## Limitations

This study had several limitations. First, the Wells-Riley model requires that the air in the room is well mixed to enable uniform distribution of aerosol, suggesting that this model considers airborne transmission but not droplet or contact transmission. Therefore, the modified Wells-Riley model with indoor CO_2_ should not be used in circumstances under where the main transmission route is droplet or contact. Second, the model is suitable only in a steady state; thus, it cannot be used in places where a lot of people come and go. Third, as the model considers the indoor CO_2_ concentration, it is also unsuitable for open spaces. Finally, although this study used a cut-off value of 1% to identify situations wherein the risk of airborne transmission hardly needs to be taken into account, further studies are warranted to validate this cut-off value.

In this study, we have estimated the airborne infection probability and R_0_ of SARS-CoV-2 Omicron strain, for we have adopted q value of Omicron provided by Dai and Zhao (2023). We were not be able to estimate the airborne infection probability and R_0_ of currently prevalent strains of SARS-CoV-2 such as XBB.1.5 and BQ.1.1. In the near future, when q value of these strains is revealed, the airborne infection probability and R_0_ are to become clear.

In the first case of aerosol infection in the pediatric outpatient room, the exposure time was set at 30 min based on the actual period of consultation. However, the pathogenic particles or aerosols emitted by the patient may have remained in the consultation room for a long time, to which the healthcare workers were exposed for longer hours. Therefore, although the infection probability in this case was estimated to be 79.7%, this may be larger in actual clinical practice as there is no way to confirm how long the pathogenic particles remain in the room. In the second case of aerosol infection, all the three patients in the ward were receiving immunosuppressants such as prednisolone, methotrexate and tofacitinib citrate for rheumatoid arthritis. Patients taking immunosuppressants are considered to be susceptible to SARS-CoV-2, suggesting that the fact that administration of immunosuppressants contributed to the establishment of infection cannot be ruled out, because the modified Wells-Riley model does not take into account the immunocompetence of susceptible individuals. In this study, we tested the validity of the modified Wells-Riley model with indoor CO_2_ in only three cases. Thus, further case accumulation or animal studies are warranted to confirm the validity of this model.

## Conclusion

In this study, we proposed a modified version of the Wells-Riley model with indoor CO_2_ studies that have reported the importance of ventilation to prevent airborne transmission and proposed useful recommendations based on the Wells-Riley model are scarce (Table 4). The present study is unique in that is suggests the application of the airborne transmission model with indoor CO_2_ in actual clinical practice.

**Table 4:** Recommendations on airborne transmission control using the Wells-Riley model

Reference	Previous report	Recommendation
Furuya 2007	Risk of transmission of airborne infection during train commute based on mathematical model	・The estimated probability distribution of the reproduction number of the influenza virus in a train had a median of 2.22 under the condition that there are 150 passengers and that 13 ventilation cycles per hour are made.
		・If the exposure time is less than 30 minutes, the risk may be low.
		・The exposure time can linearly increase the risk.
Dai and Zhao 2020	Association of the infection probability of COVID-19 with ventilation rates in confined spaces	・To ensure an infection probability of less than 1%, a ventilation rate larger than the common values (100-350 m^3^/h per infector and 1200-4000 m^3^/h per infector for 0.25 h and 3 h of exposure, respectively) is required.
		・If the infector and susceptible person wear masks, then the ventilation rate ensuring an infection probability of less than 1% can be reduced to a quarter.
Dai and Zhao 2023	Association between the infection probability of COVID-19 and ventilation: An update for SARS-CoV-2 variants	・The ventilation rates increased to ensure an infection probability of less than 1% were 8000-1400, 26000-8000 m^3^/h and 64000-250000 m^3^/h per infector for the Alpha, Delta and Omicron variants, respectively.
		・If the infector and susceptible persons are wearing N95 masks, the required ventilation rates are decreased to about 1/100 of the values required without masks.
Wang et al. 2023	Evaluation of the infection probability of COVID-19 in different types of aircraft cabins	・Flying time is the most important parameter for causing the infection, cabin types also play a role.
		・If the passengers and the index patient are not wearing masks, the infection probability will be 8% for a 10 h, long haul flight, such as a twin-aisle air cabin with a 3-3-3 seat configuration.
Authors	Present study	・In a typical outpatient setting, the required indoor CO_2_ concentration at which R_0_ is below 1 is below 620 ppm with no mask, 1000 ppm with surgical masks, and 16000 ppm with N95 masks.
		・In a typical outpatient setting, the required indoor CO_2_ concentration at which the infection probability is below 1% is below 466 ppm with no mask, 478 ppm with surgical masks, and 854 ppm with N95 masks.
		・In a typical inpatient setting, the required indoor CO_2_ concentration at which R_0_ is below 1 is below 540 ppm with no mask, 770 ppm with surgical masks, and 8200 ppm with N95 masks.
		・In a typical inpatient setting, the required indoor CO_2_ concentration at which the infection probability is below 1% is below 464 ppm with no mask, 470 ppm with surgical masks, and 654 ppm with N95 masks.

Among the suspected cases of airborne transmission, the R_0_ based on the modified Wells-Riley model with indoor CO_2_ were 3.19 in three out of the five infected people staying in the outpatient room, 2.00 in two out of the three infected people staying in the ward, and 0.191 in none o of the five infected people staying in the outpatient room. These results suggest that the modified model with indoor CO_2_ can estimate infection probability with an acceptable accuracy. In a typical outpatient setting, the required indoor CO_2_ concentration at which R_0_ does not exceed 1 is below 620 ppm with no mask, 1000 ppm with a surgical mask and 16000 ppm with an N95 mask. In a typical inpatient setting, the required indoor CO_2_ concentration at which R_0_ does not exceed 1 is below 540 ppm with no mask, 770 ppm with a surgical mask, and 8200 ppm with an N95 masks. These results make it easy to establish effective strategies for preventing airborne transmission in hospitals.

The preventive strategies for pandemic threats need to be increasingly based on efficiency, flexibility, responsiveness and resiliency to reduce the damage caused by infectious disease in society (Coccia 2021a, b). Although the modified Wells-Riley model with indoor CO_2_ has several limitations such as ignoring the effect of droplet or contact transmission and applying only a steady state, it can estimate the infection probability of SARS-CoV-2 through airborne transmission with an acceptable accuracy by measuring indoor CO_2_ concentration with a CO_2_ monitor. Organizations and individuals can efficiently recognize the risk of SARS-CoV-2 airborne transmission in a room and thus take preventive measures such as maintaining good ventilation, wearing masks, or shortening the exposure time to an infected individual by simply using a CO_2_ monitor.

## Data Availability

The datasets used and analyzed during the current study are available from the corresponding author on reasonable request.

## Appendix

**Table:**

Parameters	Value	Unit
Indoor CO_2_ concentration (C)	1116	ppm
Atmospheric CO_2_ concentration (Co)	463	ppm
CO_2_ emission rate for a person (M)	0.015078*	m^3^/h
Number of infectious individuals (I)	1	
Generation rate of infectious quanta (q)	1535	/h
Pulmonary ventilation rate for a person (p)	0.48	m^3^/h
Exposure time (t)	0.5	h
Number of individuals staying the room (n)	5	
Exhalation filtration efficacy (η_I_)	0	
Respiration filtration efficacy (η_S_)	0.5	
Infectious probability (P_I_)	79.7	%
Basic reproduction number (R_0_)	3.19	

*0.015078 = 0.01795*(0.5 + 0.9*3 + 1) / 5

The parameters of the modifeid Wells-Riley model with indoor CO_2_ in a pediatric outpatient room

**Table:**

Parameters	Value	Unit
Indoor CO_2_ concentration (C)	1187	ppm
Atmospheric CO_2_ concentration (Co)	463	ppm
CO_2_ emission rate for a person (M)	0.0099*	m^3^/h
Number of infectious individuals (I)	1	
Generation rate of infectious quanta (q)	20	/h
Pulmonary ventilation rate for a person (p)	0.48	m^3^/h
Exposure time (t)	24	h
Number of individuals staying the room (n)	3	
Exhalation filtration efficacy (η_I_)	0	
Respiration filtration efficacy (η_S_)	0	
Infectious probability (P_I_)	99.6	%
Basic reproduction number (R_0_)	1.99	

*0.0099 = 0.011*0.9

The parameters of the modified Wells-Riley model with indoor CO_2_ in a ward

**Table:**

Parameters	Value	Unit
Indoor CO_2_ concentration (C)	549	ppm
Atmospheric CO_2_ concentration (Co)	463	ppm
CO_2_ emission rate for a person (M)	0.016155*	m^3^/h
Number of infectious individuals (I)	1	
Generation rate of infectious quanta (q)	1535	/h
Pulmonary ventilation rate for a person (p)	0.48	m^3^/h
Exposure time (t)	0.25	h
Number of individuals staying the room (n)	5	
Exhalation filtration efficacy (η_I_)	0.5	
Respiration filtration efficacy (η_S_)	0.5	
Infectious probability (P_I_)	4.79	%
Basic reproduction number (R_0_)	0.191	

*0.016155 = 0.01795*0.9

The parameters of the modified Wells-Riley model with indoor CO_2_ in a respiratory outpatient room
